# Cricopharyngeal Myotomy in National Surgical Quality Improvement Program (NSQIP): Complications for Otolaryngologists Versus Non-otolaryngologists

**DOI:** 10.7759/cureus.19021

**Published:** 2021-10-25

**Authors:** Ellen M Piccillo, David Adkins, Mohamed Elrakhawy, Michele M Carr

**Affiliations:** 1 Otolaryngology, University at Buffalo Jacobs School of Medicine and Biomedical Sciences, Buffalo, USA; 2 Otolaryngology, University of Kentucky, Lexington, USA

**Keywords:** cricopharyngeus, nsqip, length of stay, surgical outcomes, surgical complications, cricopharyngeal myotomy

## Abstract

Objective: Comparing outcomes after cricopharyngeal myotomy (CM) performed by otolaryngologists (OTO) and non-otolaryngologists (NO).

Methods: A retrospective analysis of the 2014-19 ACS-NSQIP database (American College of Surgeons National Surgical Quality Improvement Program) of patients who underwent open CM (CPT code 43030) as their primary procedure. Analyzed variables include medical comorbidities, operative time, the total length of stay, readmission, reoperation, concurrent procedures, postoperative complications, and postoperative diagnoses. 183 patients were included, 97 (53%) females and 86 (47%) males. 120 had surgery by OTO and 63 by NO.

Results: There were no differences in preoperative morbidity. OTO had more outpatient surgeries compared to NO (p<.001). OTO had a longer mean operating time (p=.008). OTO had a higher proportion of concurrent laryngeal procedures and other unspecified procedures compared to NO, while NO had a higher proportion of concurrent esophageal procedures (p=.028). The total length of stay was not significantly different between the two groups. 5.8% OTO and 7.9% NO patients were readmitted for a related reason (p=.586). Complications were similar between the two groups (p>.05). NO had more postop diagnoses of acquired diverticula and achalasia of the stomach cardia, while OTO had more diagnoses of dysphagia and muscular dystrophy (p<.001).

Conclusion: There were differences in the surgical setting, length of procedure, concurrent procedures, and postop diagnoses between NO and OTO surgeons but similar complication rates.

## Introduction

Swallowing is a complex, coordinated muscular response that has a significant impact on an individual’s overall health and quality of life. Swallowing comprises of three phases: oral, pharyngeal, and esophageal, with dysfunction at any of these leading to dysphagia. Relaxation of the cricopharyngeus muscle (which sits at the junction of the pharynx and the esophagus) allows dilation of the upper esophageal sphincter (UES) and the passage of the food bolus into the esophagus [1,2]. Failed relaxation or miscoordination of this muscle is a common cause of dysphagia, and this can be attributed to central and peripheral nervous system pathology, inherent dysfunction of the muscle, and iatrogenic causes [3]. Zenker’s diverticulum is a common sequela of UES dysfunction and increased pharyngeal pressure. The diverticulum protrudes through the space between the inferior pharyngeal constrictor and the cricopharyngeus, known as Killian’s triangle [4]. Cricopharyngeal dysfunction becomes a surgical disease when dysphagia is moderate to severe and is associated with weight loss or recurrent aspiration pneumonia [4]. 

In 1951, Dr. Samuel Kaplan described the first cricopharyngeal myotomy (CM) of a patient with severe dysphagia secondary to bulbar poliomyositis. Following the procedure, the patient was able to resume oral feeding after 18 months of gastrostomy tube dependence [5]. Refinements in surgical technique and instrumentation have led to a significant reduction in the complications associated with the transcervical approach. Alternatives to traditional transcervical CM have emerged, including swallowing therapy, endoscopic balloon dilation, botulinum toxin injections, as well as endoscopic CM [3]. There has been a considerable amount of research done comparing the differences in outcomes between patients undergoing different treatment modalities for cricopharyngeal dysfunction with and without an associated diverticulum. Generally, the studies have shown that operative management, whether open or endoscopic, is superior to less invasive methods when it comes to symptomatic improvement [3,6]. Studies have been inconclusive about outcomes between endoscopic versus open surgery [3,6].

While studies have shown that CM is a relatively safe procedure with minimal morbidity and mortality, research examining operative CM complications based on patient and perioperative factors has been less common. The procedure falls within the scope of general and thoracic surgeons as well as otolaryngologists. The objective of this study is to compare complications in patients undergoing CM performed by otolaryngologists versus non-otolaryngologists.

## Materials and methods

This study involved the American College of Surgeons National Surgical Quality Improvement Program (ACS-NSQIP) public-use data files from 2014 to 2019. NSQIP includes data collected from over 800 hospitals worldwide, with over 500 in the United States. Data is collected by a certified Surgical Clinical Reviewer (SCR) from patient medical records. Case selection methods and exclusion criteria have been published previously [7]. NSQIP collects data such as patient demographics, preoperative risk factors, intraoperative variables, postoperative complications, and postoperative diagnosis. Diagnoses are presented by NSQIP as international classification of diseases-9 (ICD-9) or ICD-10 codes. The database does not include site or patient-specific identifiers. This study was reviewed by the University at Buffalo Institutional Review Board.

Patients were selected from the NSQIP database using the Current Procedural Terminology (CPT) code 43030, corresponding to open cricopharyngeal myotomy as the primary procedure. Patients were divided into two groups based on the specialty of the physician performing the surgery, either an otolaryngologist (OTO) or non-otolaryngologist, including thoracic and general surgeons (NO).

Differences between patient demographics and comorbidities were assessed to control for variation between the two sample populations. The outcomes assessed were operative time, length of stay (LOS), complications, readmissions for related reasons, reoperation for related reasons, concurrent procedures, and postoperative diagnoses. 

Data were analyzed using IBM Corp. Released 2020. IBM SPSS Statistics for Windows, Version 27.0. Armonk, NY: IBM Corp. Patient demographics, comorbidities, concurrent procedures, postoperative diagnoses, LOS, operative time, and complications were analyzed using Mann-Whitney U tests. All statistical tests assumed a significance level of .05.

## Results

Patient demographics are reported in Table 1.

**Table 1:** Patient demographics Comparison of patient demographics with regard to sex, race, inpatient/outpatient status, and age between OTO and NO groups as well as patient demographics of total patient population *Ages above 90 not recorded by NSQIP

		OTO N (%)	NO N (%)	Total N (%)	p-Value
Sex	Male	58 (48.3)	28 (44.4)	86 (47.0)	.617
	Female	62 (51.7)	35 (55.6)	97 (53.0)	
Race	Caucasian	101 (84.2)	50 (79.4)	151 (82.5)	.978
	African American	5 (4.2)	2 (3.2)	7 (3.8)	
	Asian	1 (0.8)	1 (1.6)	2 (1.1)	
	Unknown	13 (10.8)	10 (15.9)	23 (12.6)	
In/Outpatient	Inpatient	40 (33.3)	39 (61.9)	79 (43.2)	< .001
	Outpatient	80 (66.7)	24 (38.1)	104 (56.8)	
Age (years)*	Mean	70.2	71.5	70.7	.204

One hundred and eighty-three patients were included. The mean patient age was 70 years, with no significant difference in age (p=.204), gender (p=.617), or race (p=.978) between groups. Otolaryngologists were more likely to perform a CM on an outpatient basis (66.7%), as compared to NO surgeons (38.1%, p<.001). 

There were no statistically significant differences in comorbidities between the two patient groups (see Table 2).

**Table 2:** Patient comorbidities Comparison of patient comorbidities in OTO and NO groups as well as comorbidities of total patient population 1. Steroids taken within 30 days of surgery. 2. Morbidity and mortality probability is calculated from patient preoperative characteristics using logistic regression. COPD = chronic obstructive pulmonary disease, ASA = American society of anesthesiologists

Co-morbidity		OTO N (%)	NO N (%)	Total N (%)	p-Value
Diabetes	Yes	19 (15.8)	8 (12.7)	27 (14.7)	.571
	No	101 (84.2)	55 (87.3)	156 (85.2)	
Smoking	Yes	10 (8.3)	4 (6.3)	14 (7.7)	.632
	No	110 (91.7)	59 (93.7)	169 (92.3)	
Dyspnea	Yes	14 (11.6)	6 (9.5)	20 (10.9)	.660
	No	106 (88.3)	57 (90.5)	163 (89.1)	
Dependency on caregiver	Independent	114 (95.0)	60 (95.2)	174 (95.1)	.659
	Partially	3 (2.5)	3 (4.8)	6 (3.3)	
	Totally	1 (0.8)	0	1 (0.5)	
	Unknown	2 (1.7)	0	2 (1.1)	
COPD	Yes	9 (7.5)	7 (11.1)	16 (8.7)	.413
	No	111 (92.5)	56 (88.9)	167 (91.3)	
Hypertension	Yes	59 (49.2)	33 (52.4)	92 (50.3)	.680
	No	61 (50.8)	30 (47.6)	91 (49.7)	
>10% weight loss prior to surgery	Yes	5 (4.2)	3 (4.8)	8 (4.4)	.852
	No	115 (95.8)	60 (95.3)	175 (95.6)	
Steroids^1^	Yes	4 (3.3)	2 (3.2)	6 (3.3)	.954
	No	116 (96.7)	61 (96.8)	177 (96.7)	
Mortality probability^2^	Mean (95% CI)	0.004 (0.003-0.006)	0.008 (0.003-0.013)	0.006 (0.004-0.008)	.188
Morbidity probability^2^	Mean (95% CI)	0.044 (0.040-0.049)	0.048 (0.039-0.057)	0.045 (0.041-0.050)	.454
ASA class	1 -No disturbance	1 (0.8)	1 (1.6)	2 (1.1)	.054
	2 -Mild disturbance	51 (42.5)	19 (30.2)	70 (38.3)	
	3 -Moderate disturbance	67 (55.8)	38 (60.3)	105 (57.4)	
	4 -Life threatening	1 (0.8)	5 (7.9)	6 (3.3)	

Several patient comorbidities were too infrequent to warrant statistical analysis. Of the 183 patients included in the study, there were no patients on dialysis or a ventilator before surgery, no patients with disseminated cancer, sepsis, or ascites. In the OTO group, there was one patient who had received a blood transfusion within 72 hours before surgery, three patients had bleeding disorders, and three with congestive heart failure. In the NO group, there was one patient with preop systemic inflammatory response syndrome (SIRS), one with a preop wound infection, two with renal failure, and two with congestive heart failure.

There were no differences in the mortality and morbidity probabilities scores provided by the ACS. More than half the patients were in ASA Class 3 (severe systemic disease). There were no patients in ASA classes 5 or higher. There was no statistically significant difference (p=.054) between the two groups in terms of the distribution of patients belonging to each ASA class. Overall, patient comorbidities were homogenous across the two groups.

Outcome factors are displayed in Table 3.

**Table 3:** Outcome factors Comparison of outcome factors such as mean operating time, total length of stay, and total length of stay for inpatients between OTO and NO groups as well as overall outcome factors of total patient population

		OTO	NO	Overall	p-Value
Operative time (minutes)	Mean (95% CI)	85.83 (76.28-95.37)	68.87 (60.86-76.88)	79.99 (73.06-86.92)	.008
Total length of stay (days)	Mean (95% CI)	2.32 (1.47-3.16)	3.14 (1.74-4.55)	2.60 (1.87-3.33)	.289
Total length of stay (days) for inpatients only	Mean (95% CI)	4.53 (2.82-6.23) N=40	4.46 (2.27-6.65) N=39	4.49 (3.12-5.86)	.963

Mean operative time was statistically significantly longer in the OTO group, with an overall mean of 85.83 minutes versus 68.87 in the NO group (p=.008). OTO patients did not have a statistically significant difference in total length of hospital stay (2.32 days) compared to NO patients (3.14 days, p=.289). For total length of stay for inpatients only, OTO patients had an average length of stay of 4.53 days and NO patients had an average length of stay of 4.46 days, with no statistically significant difference between the two (p=.963).

Concurrent procedure data is on display in Table 4.

**Table 4:** Concurrent and other procedures Comparison of concurrent procedures such as esophageal procedures, laryngeal procedures, other unspecified procedures, and no concurrent procedures between OTO and NO groups as well as overall concurrent procedures of total patient population *5 OTO patients had both an esophageal and a laryngeal procedure and are included in both cells † Other procedures included intraabdominal procedures (2), head and neck cancer procedures (2), thyroidectomy, pharyngeal flap.

		OTO N (%)	NO N (%)	Overall N (%)	p-Value
Concurrent procedures	Esophageal procedure	26 (21.7)*	22 (34.9)	48 (26.2)	.028
	Laryngeal procedure	18 (15.0)*	4 (6.4)	22 (12.0)	
	Other †	4 (3.3)	2 (3.2)	6 (8.2)	
	None	77 (64.2)	35 (55.6)	112 (61.2)	

Of the 183 patients, 48 (26.2%) underwent an esophageal procedure during the same anesthetic event, 22 (12%) underwent a laryngeal procedure during the same anesthetic event, and six (8.2%) underwent another unspecified procedure during the same anesthetic event. All the differences in concurrent procedures were significant (p=.028). NO surgeons performed esophageal procedures in addition to a CM on a higher percentage of their patients compared to OTO surgeons (34.9% vs. 21.7%). OTO surgeons performed laryngeal procedures in addition to a CM on a higher percentage of their patients compared to NO surgeons (15% vs. 6.4%). OTO surgeons also performed a higher percentage of other unspecified procedures in addition to the CM on their patients (3.3% vs. 3.2%).

There were 12 readmissions, with seven (5.8%) in the OTO group and five (7.9%) in the NO group. This difference was not statistically significantly different (p=.586). The reasons for unplanned readmissions are listed in Table 5. 

**Table 5:** Readmissions Qualitative list of reason for readmission between OTO and NO groups

	No. of readmissions	Reason for Readmission	p-Value
OTO N=120	7 (5.8%)	Pneumonia	.586
		Shortness of breath	
		Dysphagia	
		Rash	
		Nutritional marasmus	
		Postprocedural complication (digestive system)	
		Unplanned intubation	
		Not listed	
NO N=63	5 (7.9%)	Pneumonia	
		Dysarthria	
		Localized swelling	
		Retropharyngeal abscess	
		Unplanned intubation	

The ACS-NSQIP was used to report general complications. These are presented in Table 6. 

**Table 6:** 30-day complications for patients with primary procedure cricopharyngeal myotomy from 2014-2019 Comparison of postoperative complications between OTO and NO group using NSQIP database as well as postoperative complications of total patient population (30 days) PATOS = present at time of surgery Wound dehiscence, stroke, cardiac arrest, myocardial infarction, deep vein thrombosis/thrombophlebitis, and renal insufficiency all had zero incidences. *This table includes 13 (10.8%) individual OTO patients and 10 (15.9%) individual NO patients with complications, including readmission/reoperation.

Complication	OTO N (%)*	NO N (%)*	Total N (%)
Superficial surgical site infection	1 (0.8)	1 (1.6)	2 (1.1)
Septic shock	1 (0.8)	1 (1.6)	2 (1.1)
Wound infection	1 (0.8)	0	1 (0.5)
Organ/Space surgical site infection	0	2 (3.2)	2 (1.1)
Deep incision surgical site infection PATOS	1 (0.8)	0	1 (0.5)
Pneumonia	2 (1.6)	2 (3.2)	4 (2.2)
Unplanned reintubation	1 (0.8)	2 (3.2)	3 (1.6)
Pulmonary edema	0	1 (1.6)	1 (0.5)
Ventilator >48 hours	1 (0.8)	2 (3.2)	3 (1.6)
Acute renal failure	0	1 (1.6)	1 (0.5)
Urinary tract infection	1 (0.8)	3 (4.8)	4 (2.2)
Urinary tract infection PATOS	1 (0.8)	1 (1.6)	2 (1.1)
Transfusion	1 (0.8)	0	1 (0.5)
Return to OR for related reasons	3 (2.5)	3 (4.8)	6 (3.3)
Readmission for related reasons	7 (5.8)	5 (7.9)	12 (6.6)

There were 27 reported complications by 23 individual patients, 13 OTO, and 10 NO. The most common complication in the OTO group was pneumonia (n=2), and the most common complication for the NO group was urinary tract infection (n=3). For individual complications between OTO and NO patient groups, all differences were not statistically significant (p >.05). In both groups, there were no cases of wound dehiscence, stroke, cardiac arrest, myocardial infarction, deep vein thrombosis, thrombophlebitis, or renal insufficiency. There was one death within 30 days, which occurred in an older OTO patient with a preoperative infection who developed pneumonia and sepsis. This patient also accounted for a return to OR for a related reason, for drainage of a neck abscess.

Postoperative diagnoses are presented in Table 7.

**Table 7:** Postoperative diagnoses for patients undergoing cricopharyngeal myotomy as their primary procedure 2014-2019 Comparison of postoperative diagnoses between OTO and NO groups as well as postoperative diagnoses of total patient population

Diagnosis	OTO N (%)	NO N (%)	Total N (%)	p-Value
Acquired diverticulum of the esophagus	30 (25.0)	38 (60.3)	68 (37.2)	< .001
Dysphagia	32 (26.7)	9 (14.3)	41 (22.4)	
Achalasia of cardia	13 (10.8)	11 (17.5)	24 (13.1)	
Muscular dystrophy	5 (4.2)	0	5 (2.7)	
Other	40 (33.3)	5 (7.9)	45 (24.6)	

All categories of postoperative diagnosis were statistically significantly different between the OTO and NO groups (p <.001). An acquired diverticulum of the esophagus or Zenker’s diverticulum was more common in NO patients compared to OTO patients (60.3% vs. 25%). Achalasia of the stomach cardia was more common in the NO group (17.5% vs. 10.8%). Dysphagia was more common in the OTO group (26.7% vs. 14.3%). Patients who had another unspecified diagnosis were more likely to be operated on by an OTO surgeon (33.3% vs. 7.9%). There were five patients with muscular dystrophy in the sample population and were all operated on by otolaryngologists.

## Discussion

Given the low complication and mortality rates contrasted with high rates of symptomatic improvement, CM is a safe and effective procedure for dysphagia due to UES dysfunction, with or without an associated diverticulum. Success rates, as defined by significant symptomatic improvement, are reported in the literature ranging from 75% to 100% for open surgery and 48% to 92% for endoscopic treatment [8]. Complications from CM include local hematoma, fistula, esophageal perforation, recurrent laryngeal nerve damage, and infection, most notably mediastinitis. There is a diverse range of complication rates quoted in the literature, from 0% to 25% [3,8]. In this dataset, 23 (12.6%) patients suffered complications, including 12 (6.6%) who were readmitted to the hospital and six (3.3%) who returned to the OR. Much of the morbidity and mortality associated with the procedure can be attributed to the patient population. Many patients undergoing CM are elderly, with multiple medical comorbidities, and our sample population illustrates this [8].

The groups were homogeneous other than for inpatient versus outpatient status, where otolaryngologists were statistically significantly more likely to operate in the outpatient setting. While no studies could be found relating to inpatient versus outpatient surgery and CMs, further research could be dedicated to the specific reason for this. Length of stay for both inpatients and outpatients and total length of stay for only inpatients was not statistically significantly different between the two groups. The lack of a difference between the two groups about inpatient and outpatient length of stay and total length of stay for only inpatients was expected as inpatient/outpatient status and length of stay are tracking similar aspects of patient management. There was a significant difference in length of operating time with OTO having a mean operating time of 85.83 minutes versus 68.87 minutes for NO surgeons (p=.008). The statistically significant longer mean operating time in the OTO group could be explained by the finding that the OTO patients were more likely to undergo additional unspecified procedures such as intra-abdominal procedures, head and neck cancer procedures, or pharyngeal flaps. These procedures are considerably long and could have possibly influenced the mean operating time by OTO surgeons. There were no significant differences between the NO and OTO patients concerning complications, readmissions, and re-operations. 

Regarding the findings of NO surgeons having significantly more diagnoses of acquired diverticula of the esophagus and achalasia of the cardia and OTO surgeons having significantly more diagnoses of dysphagia, muscular dystrophy, or other unspecified diagnoses, one could infer differences in surgical indications commonly encountered by the disciplines. The difference in postoperative diagnosis between OTO and NO likely reflects the differences in referral patterns and areas of focus for each specialty. An acquired diverticulum of the esophagus such as Zenker’s diverticula can be picked up via an upper esophagram ordered by a primary care physician and can be referred to their choice of surgeon. Swallowing disorders and dysphagia are complaints more commonly assessed by otolaryngologists and may represent a wider range of etiologies.

Similar studies have been performed looking at differences in outcomes of thyroid surgery between general surgeons and otolaryngologists, assessing factors such as blood loss, length of stay, vocal cord palsies, and hypocalcemia. Results highlighted differences in surgical technique and training and also revealed higher rates of vocal cord palsies in general surgery patients (4.7% vs. 8.2%, p<.001) [9,10,11].

While no one has looked at the impact of the specialty of the operating surgeon on outcomes in CM, there have been several retrospective studies looking at surgical technique and patient characteristics. Many of these studies sort patients into cohorts based on surgical indications, the presence of an associated esophageal diverticulum, and the specific technique being used. A 2007 study by Brigand et al. looked specifically at one surgeon and the 253 transcervical cricopharyngeal myotomies that he performed over 30 years, intending to identify risk factors for complications related to different surgical indications. Patients with a diverticulum were more likely to suffer from infection and fistulae, while patients with neurogenic indications, notably stroke and muscular dystrophy, experienced aspiration with subsequent pneumonia. These complications were related to airway dysfunction and not surgical technique. A mortality rate of 1.6% was reported. All four deaths were attributed to pulmonary infection in patients undergoing CM for dysphagia related to muscular dystrophy [12]. 

The inherent weaknesses of a retrospective study include the difficulty of controlling for confounding variables and preventing data entry errors. While the ACS-NSQIP database is a valuable tool for evaluating surgical care that allows for the simultaneous study of large groups of patients, data is extracted only from participating institutions. These institutions tend to include large, high volume, tertiary medical centers, introducing an inherent bias to the extracted data. Data collection is performed by different clinical reviewers at various institutions; although they are trained and certified, there is the potential for human error during data entry and extraction. Additionally, this data set is limited to the 30-day postoperative period, thereby restricting extrapolation on long-term safety and outcomes. 

The ACS-NSQIP presents several limitations specific to studying cricopharyngeal myotomies. While the database includes a postoperative diagnosis, it does not list a specific indication for surgery in each patient, and it can be difficult to fully understand the clinical picture. Many of the postoperative diagnoses are general. For example, dysphagia and acquired diverticulum of the esophagus are often included in our sample. It’s possible that a patient with a diverticulum presented with dysphagia, which was listed as the postoperative diagnosis. While the database does comment on the presence of a Zenker’s diverticulum, it fails to comment on the size or associated symptoms, which are important criteria when planning management. The database does not include any variables indicating surgical success or symptom improvement; these are outcomes, and the database is geared to examine complications. This database is not intended to be used to compare treatment efficacy. When analyzing concurrent procedures, it is important to consider that CM may be performed as a component of a more extensive surgery such as a laryngectomy. While our analysis of NSQIP only included cases where CM was listed as the primary procedure, this does create a potential point of error for data collectors that would disproportionately affect OTO patients. Finally, this database is geared towards general surgical complications, so clinical information about CM-specific complications is limited (for example, fistula formation, aspiration, or recurrent laryngeal nerve palsy).

## Conclusions

The NSQIP database suggests that CM is a low-risk procedure. There may be differences in inpatient versus outpatient setting, length of procedure, concurrent procedures performed under the same anesthetic event, and postoperative diagnoses. OTO surgeons tend to perform more outpatient surgeries, with different concurrent and longer procedures performed compared to NO surgeons. OTO surgeons also had significantly more postop diagnoses of dysphagia and muscular dystrophy compared to NO surgeons who had more postop diagnoses of acquired diverticula of the esophagus or Zenker’s diverticulum and achalasia of the stomach cardia. Procedure-specific multi-institutional databases have value, but this study points out that single specialty databases may have limitations. Because CM is an uncommon procedure performed by multiple surgical specialties, it would be appropriate to develop an arm of NSQIP devoted to complications specific to CM. 
